# Supplementary material for: Sensitivity of discrete symmetry metrics: Implications for metric choice
Source: PLoS One. 2022 May 19;17(5):e0268581. doi: 10.1371/journal.pone.0268581 (PMC9119531; doi:10.1371/journal.pone.0268581)
Supplement: Attachment — Submitted filename: response-to-reviewers.docx [file pone.0268581.s001.docx]

We would like to thank the Editor and Reviewers for the time they took to read through our manuscript and provide us with their valuable feedback. We have incorporated most of the feedback provided to us during the review process or provide clear rational when it was not possible. Please see our point-by-point responses to each comment below.

Sincerely,

Allen Hill and Julie Nantel

Reviewer #1: Main feedback to the author

The authors have investigated the sensitivity of different discrete symmetry metrics available in the literature. The topic is of interest to help researchers, clinicians, and other professionals of interest to investigate symmetry as, for example, a recovery indicator based on a sound and robust methodology. In this study, the authors have first considered a theoretical approach to investigate the sensitivity of the included discrete symmetry metrics. In a second step, gait data was considered to experimentally verify the robustness of the discrete symmetry metrics investigated. This is a very elegant and valid approach. However, considering the broad range of signals available in both healthy and pathological populations exhibiting asymmetric characteristics, restricting the analyses only to bilateral signals with the same sign, e.g., positive, potentially is not the most representative approach. The current analyses exclude relevant signals, e.g., kinetic data, which could contribute to understanding better the asymmetry in multiple populations. I strongly suggest the authors reconsider this and the aspects described below to improve the manuscript quality.

The following comments need to be addressed:

Major issues

Line 114: I would appreciate it if the authors could comment on the definition of R = [1,5]. This approach only includes variables with the same sign (x and y > 0, for example), which is not fully representative of kinetic data, for example. I would agree with this definition if the authors would only be interested in spatiotemporal aspects (or any other signal only with positive OR negative signs). If this is the case, the type of signal of interest must be referred to on line 96, i.e., x and y are considered to always have the same sign. However, if the authors choose this strategy the usability of this manuscript will be limited and it should be included in the limitations. I would suggest including another range that crosses zero and experimental data that reflects it (e.g., ground reaction forces in the anteroposterior direction, which may be used in stroke populations – the authors refer to this population in line 38).

- We thank reviewer #1 for their thorough and insightful review of our manuscript. We acknowledge and agree that our current approach, by restricting inputs to have the same sign, cannot representative of all data, such as kinetic data which may feature opposite signs. This was an intentional choice and is fundamental to our analysis. A clarification was added to the methods section to denote this. The sentence now reads:
  - Line 98: “We assume that the inputs x and y are independent, normally distributed (an assumption made by many parametric tests), have the same sign, and have the same shape…”
- Several factors contributed to our choosing of this restriction. Our analysis is structured around representing the asymmetry of the data as a ratio between the two random variables which form the arguments to the generic symmetry function S(x,y). This allows comparing different symmetry functions evaluated on data of the same underlying asymmetry magnitude. However, a ratio (y/x) has a vertical asymptote at x=0, where $\lim_{x\to+0} \frac{y}{x}=+\infty$ and $\lim_{x\to-0} \frac{y}{x}=-\infty$. This asymptote causes problems numerically in the power simulations (in terms of the code design, and for the several metrics that include ratios in their definitions), and in analyzing/presenting the data. Finally, we will note that the Alves et al. (2020) paper performed somewhat similar comparisons and validation using kinetic data, including data which featured opposite signs. However, it is not clear to us that “asymmetry” as a concept is even appropriate for bilateral variables that may feature opposite signs, but this is somewhat orthogonal to the aims of our paper.

General comment for the results section: it is difficult to follow the figures and the results section with the multiple references and labels used in both the text and the figures’ captions. Please be consistent: either use the abbreviations you have in the figures or use the references in the figures. I would strongly advise using abbreviations as a simple and efficient solution. This should ideally be introduced in the Methods section (line 104). Please also refer to the figures’ panels (e.g., Figure 1 A) when describing the results within the text.

- Abbreviation definitions have now been added to the manuscript (immediately prior to first naming of metrics in Table 1) and used throughout the results and discussion section where appropriate.
  - Line 153: “Symmetry metrics are abbreviated as the first three letters of the first author followed by the last two digits of the publication year, and metrics with an absolute value are distinguished by an absolute value function annotation, e.g. “abs(Plo05)”.”
- Additional references to figures and specific panels have been included in the text where and when appropriate. We note that the figures do not display all of the results of the simulation due to the magnitude and complexity (4 variables/dimensions). Therefore, several sentences in the results describe specific behaviors not solely apparent in the figures.

Line 273: I recommend caution with this statement since the authors did not investigate other types of signals. It is currently unknown what would happen in scenarios that lead to “artificial inflation”. Furthermore, considering the results described in lines 163 – 166, this recommendation could mislead future results comparisons.

- We have rephrased this sentence from a suggestion to presenting it as an option for those who wish to benefit from more sensitive metrics while maintaining the intuitiveness of ratios.
  - Line 269: “To improve the communication of results while maintaining a higher power for statistical testing, a metric with good sensitivity could be used for the statistical analysis, and then the results of the analysis—means and confidence intervals—could be transformed to ratios for reporting; such an approach would combine the strengths of a more sensitive metric and the intuitiveness of a ratio.”
- We agree that without sufficiently explicit description, this approach could be misleading regarding which metrics were used and how. However, for purposes of comparing results, scalar values (i.e. single values, such as mean asymmetry) can be exactly converted between any of the metrics in this paper, including a “round trip” conversion, e.g. converting a Rob87 metric to ratio and back would end with the same asymmetry in Rob87 “units” as the original value. Our code (available in Zenodo) includes the capability to do convert between asymmetry metrics assessed in this paper.
- “Artificial inflation”, as termed by Alves et al. (2020), would not be a concern in this situation, as it is only an issue (in terms of obscuring legitimate effects) when performing the statistical tests and our recommendation is to only use the ratio for presenting results, not for statistical tests. Additionally, by transforming only scalar values (mean and upper, lower CI bounds), the high variability causing the “artificial inflation” would be limited and controlled by whichever more sensitive metric was used in the actual analysis.

Minor issues

Line 45: While I understand what the authors refer to, I would suggest rethinking the use of "discrete symmetry metric". Potentially, a reference to “method” instead of “metric” could improve manuscript readability. Why did the authors use the abbreviation DSM only in the introduction? I would suggest using DSM across the whole text or using only the long-form.

- We acknowledge that “method” and “metric” are nearly synonymous, and the choice comes down to personal preference. We believe that “method” may subtly suggest a more fundamentally different approach than “metric”, which to us refers to the more limited differences between the symmetry metrics, which are only distinguished by defining equation, but are otherwise fundamentally the same.
- We limited use of DSM to the introduction because there was also limited discussion and contrast against continuous symmetry metrics. However, our stated purpose at the end of the introduction is to only analyze the behavior of DSM, therefore, continuing to clarify that we were referring to “discrete” symmetry metrics through the rest of the paper was deemed superfluous.

Line 104: Please refer to the discrete symmetry metrics, potentially with abbreviations as done in the figures’ captions. This will help the manuscript's readability.

- We have added the description of the metric name abbreviation format in the Results section and introduced the abbreviations in Table 1, immediately prior to the first instance of referencing metrics individually by name. We believe that introducing the abbreviations earlier, in the sentence on line 104 would be premature and detract from the clarity of the point of that section, which was to initially describe inclusion criteria and cite the papers which published the metrics to be included.

Lines 149 – 151: Please specify in which discrete symmetry metrics the order of x and y was adjusted.

- We understand the desire for greater specificity, however, the many different scenarios in which metrics were defined (and used in practice) make this impractical/unclear. Nearly all metrics were not originally defined with generic “x” and “y” inputs. For example, the Sel86 metric was defined between sound and prosthetic legs in a study of people with transtibial amputation. Similarly, the Rob87 metric used injured vs uninjured, the Vag92 metric used left and right, the Plo05 used functionally defined “shorter swing time” and “longer swing time”. Additionally, these metrics have been used elsewhere in the literature with different populations/contexts than the original. Therefore, there is not a clear and consistent relation to map the generic “x” and “y” used in our study to the original bilateral inputs. The goal of our clarification on line 149-151 was to communicate that we have ensured that our presentation of the equations was internally consistent, such that a larger “y” than “x” resulted in a larger (positive) asymmetry value for every metric.

Lines 158 – 160: I assume the authors are comparing Figure 1 A to Figure 1 B and Figure 1 A to Figure 1 C. However, it would be helpful if the authors could guide better the reader through this result. This is a relevant figure to understand the sensitivity of the different discrete symmetry metrics and the discussion/findings.

- You are correct, Fig 1A vs 1B, and Fig 1A vs 1C. We have included these figure references and comparisons in the referenced sentence. We think these changes sufficiently address this comment, however, we are unsure if this reviewer is requesting additional discussion/guidance in the text of the results, beyond clarifying the comparison between figure panes.
  - Line 163: “As sample size increases and group/condition variability decreases, all metrics reach sufficient power at a lower asymmetry magnitude (Fig. 1A vs 1B, 1A vs 1C).”

Line 163: please be consistent with the range you report. A range of [0,1] (in figures for example) and [0,100] may represent the same but it should be reported consistently.

- We humbly suggest that the reviewer may have misread the citation for the Seliktar and Mizrahi (1986) paper, reference 10, as a range (“[10]”)? Line 163 did not contain a range. The implementation of metric abbreviations reduced the repetition of in-text citations to papers and has removed the reference which may be the cause for the misreading.
- Alternately, based on the reviewer’s choice of 1 and 100, they may have been referring to the nearby mention of large sample size (N=100) or variability (σ=1.0) on line 165. These did not refer to ranges, nor are they equivalent. The intent of the sentence was to communicate that a large variability (σ=1.0) can be detrimental to power, even when sample or effect size (i.e. asymmetry magnitude) are optimal (i.e. very large, including large sample sizes of N=100, the largest simulated).

**(NB: The following 3 comments were rearranged to respond to as a group.)**

Line 164: why did the authors use a value on line 164 (0.25) and another in the figures (0.32)?

Lines 164 – 166: where can the reader see this result? It would be helpful if the authors could explain this better.

Lines 175 – 176: where can this be observed in Figure 1? The three panels refer to variability greater than 0.25.

- The complete results of the simulation encompass 4 dimensions (metric, ratio, variability, and sample size) and are not feasible to fully communicate graphically or via tables. Therefore, simulation results have been summarized using figures where possible, while key individual behavior (i.e. distinct and relevant changes at certain sets of parameters) were communicated only in the text of the results. We have clarified our mention of the availability of simulation code at the end of the discussion to note that the full simulation results are also present in Zenodo.
  - Line 256: “Our simulation code and results are available in Zenodo and can be explored or expanded to test characteristics or assumptions not made here [27].”
- Additionally, our intent for Figure 1 was to highlight the different effects of variability and sample size at moderate values that still demonstrate practically significant changes in behavior. However, the sentence on line 164-166 communicates an important change in the behavior of the ratio metric, which occurred at a smaller variability than shown in Fig. 1. While the variability .25 could have been used in Fig. 1, size/scale and resolution considerations dictated that a larger variability, where the change in behavior of the ratio became more pronounced, was a better choice for visual clarity. The sentence on 175-176 is similarly describing an important behavior that is not apparent from the figures.

Lines 166 – 168: I assume that “the absolute value Plotnik et al. [13]” refers to abs(Plo05). From what I can see, abs(Plo05) did not reach 100% power for all variabilities (Figure 1 A).

- You are correct, “reach” was a typo that has been replaced with “approached”.

Lines 168 – 170: How can we see this effect (slow increase in power) in Figure 2 if Figure 2 refers to “Minimum sample size to achieve sufficient power (80%)”? Is this not supposed to be read together with Figure 2?

- We have added a reference to Fig. 1 as well. However, Fig. 2 is a more thorough demonstration of this point. While Fig. 1 shows increases in power with increasing asymmetry magnitude, Fig. 2 effectively shows increases in power with sample size, where a faster increase in power will result in a smaller minimum powered asymmetry magnitude (lower on the Y-axis). The Sel86 and abs(Plo05) metric lines are distinctly higher on the Y-axis, e.g. larger minimum powered asymmetry magnitude, than the other metrics. Fig. 1 shows this slower increase in power for only 3 sets of variability and sample size, while Fig. 2 shows it for all sample sizes for 2 simulated variabilities.

Lines 168 – 170 and 173 – 175: The authors report in the results section that some discrete symmetry metrics increase in power faster than others. Considering this, I would expect the authors to have discussed this in this section. What could be consequences (and even recommendations) following such a result?

- We used the term sensitivity to generally refer to the rate of increase in power. Although increasing in power faster is an important consideration, robustness against variability is another relevant factor to consider. Our discussion comments on the differences in sensitivity we observed (lines 209-215), highlights the poor sensitivity of the ratio (lines 238-242), and recommends the use of the most sensitive metrics (lines 243-252, and the Recommendations section). In particular, we note the consequences of choosing a metric with poor sensitivity, using the ratio as an example, on line 242. The consequence discussed there, large variation obscuring significant results, is effectively the “artificial inflation” referred to by Alves et al. (2020). However, we disagree with their interpretation of the large variation as “artificial”, as technically the large variation is the correct result, from an analytical consideration of the mathematics and probability. Instead, such large variation could suggest an excessively heterogenous or non-uniform population that a study design didn’t account for (e.g. low power due to insufficient sample size, or sub-groups with unique characteristics).

**(NB: The following 2 comments were rearranged to respond to as a group.)**

Line 192 – 193: throughout the manuscript, the reader can read “swing time proportion”, “swing time percent” (line 135), and only “swing time”. Keeping it consistent would help the manuscript’s readability.
Line 202: Swing time (%) proportion asymmetry or swing time (s) asymmetry? Please be consistent throughout the manuscript.

- We have removed any uses of swing time “proportion” and limited it to swing time percent. Additionally, we added a sentence in the methods to explain that swing time is only used in units of percent in this paper, therefore further mention of swing time will avoid repeating the units.
  - Line 136: “Swing time was calculated in units of percent stride; future references to swing time will omit mention of the units.”

Lines 197 – 198: the authors did not report swing time asymmetry evaluated with all metrics in Table 3. The mean difference was reported. Please keep this consistent and explain what the mean difference refers to.

- Mean difference refers to the mean difference between tied- and split- belt conditions. The actual asymmetry values for each metric were not reported, as they were not relevant to purpose of the study, which is effectively to compare the relative effect sizes (which is directly related to power, as described in the manuscript). Therefore, the mean difference, confidence intervals, and effect sizes were deemed relevant and most equivalent to the simulation in terms of contributing to core points, while the actual asymmetry values would not have contributed to core points.

Figures: the figures are beautiful. Consistent, good choice of colors, and appropriate size. I like very much the strategy to aggregate the discrete symmetry metrics with very similar results – it improves the figure a lot! I only have very minor suggestions:

Figure 1: It would be very helpful if the authors could include in each panel the sample size and the variability used.

Figure 2: It would also be very helpful if the authors could include in each panel the variability used.

- We have added text to the panels in Figures 1 and 2 including the sample size and/or variability, as appropriate.

Table 3: Expanding the captions would be of great help for the reader. Your paper could be better understood, in my perspective. For example, briefly describe what mean difference refers to.

- We have added a description of mean difference for Table 3.

Lines 209 – 215: this is results repetition (lines 160 – 163, for example) – all valid and important results. Considering the large number of investigations that are based on some of the discrete symmetry metrics tested in this study that had poor sensitivity (e.g., the ratio [10]) or similar sensitivity (e.g., Robinson et al. [11], Zifchock et al. [14]), the authors could expand this.

- While this paragraph is mildly repetitive, as it represents the core results of the paper, we believe it should be succinctly summarized and highlighted at the beginning of the discussion.
- Additionally, we wish to be clear that the intent of our paper was not to produce strict guidelines regarding metrics which should or should not be used. Instead, our goal was to provide a comprehensive review of the behavior regarding sensitivity of symmetry metrics, along with our interpretation of the results, and allow the readers to reach their own conclusions regarding the suitability of metrics for their own specific needs and context.

Lines 243 – 244: from what I can observe in Figure 2, Rochester et al. [16] is not the most sensitive. Rochester et al. [16] and the weighted Alves et al. [18] have the same sensitivity. I could also not find this result before. Please reconsider either here or in the results section to keep consistency.

- A sentence was added following the sentence in question acknowledging that the weighted Alv20 metric is equivalent.
  - Line 241: “The weighted Alves et al. [18] metric is a normalized alternative that has practically equivalent sensitivity.”

Line 262: What do the authors mean by “continued use of multiple symmetry metrics”? To use multiple discrete symmetry metrics without considering their sensitivity? This would be opposite to lines 272 – 273.

- We have rephrased that sentence as follows to better convey the message that there is no clear “best” overall metric:
  - Line 259: “The results of this study show that multiple symmetry metrics demonstrate sufficient sensitivity for a broad range of data”
- We are, of course, not advocating for the use of metrics without considering their sensitivity, instead, we acknowledge that several metrics have practically equivalent sensitivity, such that one cannot choose a metric purely based sensitivity.

Reviewer #2: The authors verified the sensibility of metrics employed by the literature to calculate gait (a)symmetry. The study has the potential in supporting further studies in selecting the most appropriate metrics to examine gait asymmetry. Although, in my opinion, the study might be relevant, I think some segments of the manuscript should be strengthened. For instance, even though it is not the intention, the authors should explain in more detail the clinical/functional relevance of studying gait asymmetry. Mainly, what I am struggling with is the end segment of the study, since, considering the title (“Sensitivity of discrete symmetry metrics: implications for metric choice”), I was expecting a recommendation in terms of the most appropriate metric to select to calculate gait symmetry. If it is not possible because metrics may have pros and cons, I would recommend a list mentioning these pros and cons aspects to guide future research in the field.

Specific comments

- Introduction - Across paragraph 1

The authors introduced the topic and briefly discussed clinical aspects of gait asymmetry. I believe it is possible to strengthen by including the clinical and functional relevance of examining gait asymmetry. Perhaps these questions might help: In gait study, what is the implication of gait asymmetry? If gait asymmetry is implicit in humans, why is important to reduce gait asymmetry in the neurological population? Also, briefly, what are the physiological, neural, and/or biomechanics underlying mechanism related to gait asymmetry that makes this topic relevant to study?

- We thank reviewer 2 for their time and feedback in review of our manuscript. We agree that the mentioned topics (implication of gait asymmetry; physiological, neurological, and biomechanical mechanisms; and value in reducing asymmetry) are important and interesting topics to consider, but they are outside the scope of this study: The purpose of this paper is to inform high-powered study design involving assessments of gait asymmetry, regardless of the specific population or research question, beyond an assessment of gait asymmetry. Additionally, such discussions already exist, and a few are cited in the introduction of our manuscript.

Introduction paragraph across lines 48-59

- I am confused with the information in this paragraph;

1. the authors referred to 5 discrete methods. However, if I understood it correctly, there were 8 described metrics (DSMs) (Refs. 10, 11, 12, 13, 14, 15 and 16, 17 and 18) So, was it 5 or 8, or Did I miss something?

- Five historically common metrics are mentioned in the introduction, however, as stated in the methods section, lines 105-111, 3 more metrics were included in our analysis, for a total of 8. Throughout the methods, results, and discussion, 8 metrics are present and discussed.

2. As the information is described in this paragraph, I could not understand whether there are differences and overlaps between the metrics. I recommend the authors review the paragraph clarifying in more detail what made the metrics different. I

- The different DSM discussed in this paragraph are all conceptually equivalent and all overlap, which is the basis for the value that this manuscript provides, by providing an evidence based discussion of the differences between these DSM that could inform choice of a DSM. To improve clarity, the final sentence of this paragraph was changed in the manuscript as follows:
  - Line 57: “Despite their conceptual equivalence, the different defining equations for all of these DSM produce unique numeric results which are not directly comparable [19].”

3. The authors should call table 1 here and maybe include more information in the table to discriminate or link the information of the metrics

- Including Table 1 in the introduction would be unclear for many readers, however, without the context of the entire introduction and the methods section, the relevance of many details in Table 1 would not be immediately clear. For example, although the equation definitions could support our introduction of differences between symmetry metrics, the inclusion at that point would add unnecessary detail and suggest a stronger focus on the equations, when the actual focus of this paper is on power and metric sensitivity. Therefore, we feel that an earlier placement of Table 1 would result in undesirable confusion.

Methods, lines 106 – 108 “Two metrics [13,16] are defined with absolute values and solely assess asymmetry magnitude; to enable consistent comparisons between metrics, these two metrics were assessed with and without the absolute value applied.”

- I believe being important to explain better this segment, meaning what did the authors mean to say using absolute value and magnitude? how do the studies differentiate that?

- Absolute value refers to the mathematical function which removes the sign (and therefore directionality, e.g. left is larger than right) and only conveys the magnitude (i.e. size) of the asymmetry (e.g. there is a bilateral asymmetry of magnitude X, but the directionality, whether left or right is larger, cannot be determined from X). This is different from all other metrics where the sign of the result (positive or negative) conveys the direction of the asymmetry (eg left or right is larger).
- As discussed in the manuscript, all but 2 metrics were directional, as published. However, in practice, authors may sometimes apply an absolute value to the results from directional metrics if asymmetry direction is not relevant to their research question.

Experiment results

- If it is possible, as the authors addressed the relevance of power, I strongly recommend the authors to include a column in tables indicating the current power for the comparisons.

- We believe that the reviewer is referring to post-hoc power. However, this would not be informative or statistically sound [1-3]. In brief, power is the likelihood to detect a difference, if one exists. After an experiment and statistical tests have been performed, the presence or absence of an effect is fixed. In the case that there is an effect, if the tests returned a significant result, then the functional “post-hoc” power is 100%*. In the case of an effect that was not detected (non-significant test result), that would be a false negative (Type II error). However, in most cases for experimental data, it is impossible to know *de novo* whether an effect exists or not. This is the reason statistical tests are performed, and the reason for the extensive power simulations in our study: power cannot be determined for experimental tests. Statistical power is only useful for planning purposes to maximize the likelihood a study will be able to detect an effect if one exists.
  *Post-hoc power is actually directly calculated from the p-value, where any significant p-value (regardless of critical alpha) crosses a power of 0.5 at the critical alpha.

  1. Hoenig JM, Heisey DM. The Abuse of Power. Am Statistician. 2001;55: 19–24. doi:10.1198/000313001300339897
  2. O’Keefe, D. J. Brief Report: Post Hoc Power, Observed Power, A Priori Power, Retrospective Power, Prospective Power, Achieved Power: Sorting Out Appropriate Uses of Statistical Power Analyses. Commun Methods Meas 1, 291–299 (2007).
  3. Althouse, A. D. Post Hoc Power: Not Empowering, Just Misleading. J Surg Res (2020) doi:10.1016/j.jss.2019.10.049.

Recommendation sections – Discussion

- In my opinion, as a recommendation, I suggest including a bulleted list indicating the main points found by the authors. This could help guide further study in gait asymmetry.

- A summary of our results is included as the first paragraph of the discussion, and main points are found on lines 239-249, as well as the conclusion. We would prefer to leave the structure of the discussion as written, and it is not clear to us whether such formatting would be acceptable in PLOS One.

Discussion

Somehow, to me, it would be important to readers if the authors could include some discussion about the clinical implication of selecting one or another metric of gait asymmetry

- It is important to us that clinicians consider the needs of their particular study when choosing a symmetry metric. While we have included recommendations for two metrics with excellent sensitivity, unique needs may require a clinician to choose another metric based on a consideration of the results presented in our paper. We therefore endeavored to provide a comprehensive evaluation of multiple symmetry metrics using a broad range of parameters to maximize the information available to authors designing a study which includes an assessment of gait asymmetry. Additionally, as we have noted in the introduction, these various symmetry metrics are functionally equivalent, therefore, the primary clinical implications in considering multiple symmetry metrics will be the likelihood of detecting an effect, if one exists, i.e. the sensitivity.

Discussion

- I recommend including a limitation subsection. I noticed that the authors described some limitations across the discussion, but I strongly recommend summarizing the limitation in a specific section.

- Limitations of this paper are only discussed in the final paragraph of the discussion; therefore, we don’t believe that adding a sub-heading would significantly add to the clarity, as the limitations will only make sense after reading the preceding sections of the manuscript.

Conclusion

- In my opinion, the conclusion needs to be improved in terms of indicating the most appropriate metric(s), as it was implicitly mentioned in the title.

- We have added a sentence to the conclusion to mention the two most sensitive metrics found in our results. However, the “implications for metric choice” noted in the title refers to the general finding that not all symmetry metrics have equivalent sensitivity, and therefore, researchers should consider power and sensitivity when choosing a metric to assess gait asymmetry.
