# Supplementary material for: Sensitivity of discrete symmetry metrics: Implications for metric choice
Source: PLoS One. 2022 May 19;17(5):e0268581. doi: 10.1371/journal.pone.0268581 (PMC9119531; doi:10.1371/journal.pone.0268581)
Supplement: Attachment — Submitted filename: Response_R2.docx [file pone.0268581.s002.docx]

We thank reviewer 2 for their continued dedication to improving the content and communication of our study. We have accepted (with a minor rephrasing) the suggested modification to the sentence (line 280-281) in the conclusion. The sentence now reads as “Two metrics, published by Rochester et al. [16] (when used without an absolute value) and Alves et al. [18], display excellent sensitivity to a broad range of data characteristics.”

Sincerely,

Allen Hill and Julie Nantel
